# Characteristics of Cognitively Unimpaired Older Adults with Motoric Cognitive Risk

**DOI:** 10.1002/alz.084676

**Published:** 2025-01-03

**Authors:** Stephanie E. Large, James Hall, Sid E. O'Bryant

**Affiliations:** ^1^ University of North Texas Health Science Center, Fort Worth, TX USA

## Abstract

**Background:**

The concept of motoric cognitive risk (MCR) combines subjective cognitive concern (SCC) with slowed gait speed. The concept allows for the incorporation of cognitive and functional slowing into a measure of risk assessment. This study explores differences in cognitive functioning in cognitively unimpaired older adults with MCR and those without subjective cognitive concern and without slow gait speed.

**Method:**

Data from 310 cognitively unimpaired participants from an epidemiological study of aging was collected. The MCR was defined as 1 standard deviation above the mean (controlled for age category and gender) for a four‐meter gait speed walk test and the report of SCC. Then each participant with MCR was matched with an equivalent non‐cognitively impaired participant with no SCC and matching for age, gender, and education. Two participants with MCR were excluded due to not having a match. T‐tests were performed comparing performance on the Geriatric Depression Scale (GDS) and raw scores Wechsler Memory Scale III sub tests Logical Memory I, II and Digit Span, Spanish English Verbal Learning Test (SEVLT) and Trail Making Test Part B.

**Result:**

308 participants were included in the study with a mean age of 65.04 (8.4), mean education of 11.10 (4.72). The results showed significant differences between the MCR and no MCR groups in on the GDS t (306) = 7.99, p <.001, SEVLT immediate t (306) = ‐5.05, p < .001, SEVLT delayed t (306) = ‐4.84, p <.001, Logical Memory I t (306) = ‐3.79, p <.001, Logical Memory II t (306) = ‐4.18, p < .001, Trail Making Test Part B t (285) = 3.05, p = .003. The digit span sub test was not significant.

**Conclusion:**

The study results demonstrate a significant difference in cognitive test performance and depression between those with MCR and without. This supports MCR as a distinct group and potentially as a tool to identify those at risk for later cognitive decline. Future research should examine the relationship between depression and MCR to determine causality, ATN markers in MCR and utility of MCR as a diagnostic category.

